# Small-Molecule Inhibitors (SMIs) as an Effective Therapeutic Strategy for Endometrial Cancer

**DOI:** 10.3390/cancers12102751

**Published:** 2020-09-24

**Authors:** Cristina Megino-Luque, Cristian Pablo Moiola, Clara Molins-Escuder, Carlos López-Gil, Antonio Gil-Moreno, Xavier Matias-Guiu, Eva Colas, Núria Eritja

**Affiliations:** 1 Oncologic Pathology Group, Department of Basic Medical Sciences, Biomedical Research Institute of Lleida (IRBLleida), University of Lleida, Av. Rovira Roure 80, 25198 Lleida, Spain; crismegino@cmb.udl.cat (C.M.-L.); cmolins@irblleida.cat (C.M.-E.); fjmatiasguiu.lleida.ics@gencat.cat (X.M.-G.); 2 Centro de Investigación Biomédica en Red de Cáncer (CIBERONC), Monforte de Lemos 3-5, 28029 Madrid, Spain; agil@vhebron.net (A.G.-M.); eva.colas@vhir.org (E.C.); 3 Gynecology Department-Biomedical Research Group in Gynecology, Vall d’Hebron Research Institute (VHIR), Universitat Autonoma de Barcelona, Pg. Vall d’Hebron119-129, 08035 Barcelona, Spain; carlos.lopez@vhir.org; 4 Laboratory of Precision Medicine, Oncobell Program, Bellvitge Biomedical Research Institute (IDIBELL), Department of Pathology-Hospital, Universitari de Bellvitge, Gran via de l’Hospitalet 199, 08908 Barcelona, Spain; 5 Oncologic Pathology Group, Department of Medicine, Biomedical Research Institute of Lleida (IRBLleida), University of Lleida, Av. Rovira Roure 80, 25198 Lleida, Spain

**Keywords:** endometrial cancer, small-molecule inhibitor, PI3K/AKT/mTOR, receptor tyrosine kinase, clinical trials

## Abstract

**Simple Summary:**

Patients diagnosed with endometrial cancer (EC), the most common gynaecological malignancy in women worldwide, cope with a disease associated with poor prognosis and limited treatment options after first-line therapy when it reaches an advanced or metastatic stage. Lately, small-molecule inhibitors have emerged as an alternative targeted therapy, renewing hope in the fight against this disease. The aim of this review is to shed light into the current state and future prospects of small-molecule inhibitors on EC treatment by summarizing the extensive number of clinical trials that have been performed during the last years, and to provide a comprehensive up-to-date document with the most remarkable results. Despite the great effort researchers are making to improve the molecular characterization of tumours, to unravel the underlying mechanism of EC progression, and to increase the efficacy of targeted therapy, we might say that there is still a long way to pave to efficiently treat EC patients.

**Abstract:**

Endometrial cancer (EC) is the sixth most common cancer in women. A continued number of low-risk EC patients at diagnosis, as well as patients diagnosed with advanced-stage disease, will experience an aggressive disease. Unfortunately, those patients will present recurrence or overt dissemination. Systemic cytotoxic chemotherapy treatment on advanced, recurrent, or metastatic EC patients has shown poor results, with median survival rates of less than one year, and median progression-free survival rates of four months. Therefore, the search for innovative and alternative drugs or the development of combinatorial therapies involving new targeted drugs and standard regimens is imperative. Over the last few decades, some small-molecule inhibitors have been introduced in the clinics for cancer treatment, but only a few have been approved by the Food and Drug Administration (FDA) for EC treatment. In the present review, we present the current state and future prospects of small-molecule inhibitors on EC treatment, both alone and in combination.

## 1. Introduction

Endometrial cancer is the most common gynaecological malignancy and the sixth most occurring cancer in women worldwide. In terms of mortality, EC accounts for almost 90,000 deaths worldwide every year [1]. The disease is usually diagnosed in its early stages and associated with good prognosis, but approximately 20% of patients will present regionally extensive disease and 8% of them will have distant metastasis. In addition to tumour spread, measured through the International Federation of Gynaecology and Obstetrics (FIGO) staging system, other prognostic factors include histological type, histological grade, and lymphovascular space invasion (LVSI). Since 1983, EC has been classified using a dualistic classification: Type I or endometrioid subtype, which mostly includes the endometrioid histology and is associated with good prognosis; and Type II or non-endometrioid EC, which includes different minor histologies, such as serous, clear cell, mixed cell adenocarcinoma, carcinosarcomas, and other rare types, and is associated with poor prognosis [2]. In 2013, The Cancer Genome Atlas (TCGA) Research Network proposed a molecular classification that differentiates four groups of ECs: Polymerase ε (POLE) ultramutated, microsatellite stability unstable (MSI) hypermutated, copy-number low (microsatellite stable, MSS), and copy-number high (serous-like) [3]. Most of the endometrioid ECs subtypes that were classified with the dualistic classification are now classified into the POLE ultramutated, MSI hypermutated, and MSS groups, while few p53 (also known as TP53) mutated endometrioid EC and all serous EC tumours are now grouped in the serous-like group. The POLE group is characterized by having an extraordinarily good prognosis, even though it includes some high-grade tumours, and the serous-like group is the one with the worst prognosis [4]. 

The cornerstone treatment of EC is surgery, complemented with radiotherapy and chemotherapy upon the presence of poor prognostic factors. Unfortunately, for diagnosed women with poor prognostic factors who experience a recurrence, or develop metastatic disease, treatment options are limited. While platinum-based regimens are often administered as adjuvant therapy, this treatment has shown poor results, with median survival rates of less than one year, and median progression-free survival (PFS) rates of four months in this subgroup of patients [5]. Therefore, the search for innovative and alternative drugs or the development of combinatorial therapies involving new targeted drugs and standard regimens is imperative.

Over the last few decades, small-molecule inhibitors (SMIs) have gained acceptance as new therapeutic alternatives in cancer, since they offer a targeted approach to treatment and are expected to have fewer adverse side effects than current chemotherapy regimens. Small-molecule inhibitors are compounds of less than 500 Da in size and are often administered orally. They are developed to target any portion of a molecule, regardless of the target’s cellular location [6]. Indeed, there are SMIs that target both extracellular and intracellular proteins, such as cell surface ligand-binding receptors and anti-apoptotic proteins, among other protein types. Research on small-molecule drugs has been so far successful. Despite several drugs having been introduced in the clinic for cancer treatment, only a few have been approved for EC treatment. The present review aims to compile clinical studies that have been performed in relation to EC to evaluate the effectiveness of SMI, which targeted the phosphatidylinositol-3-kinase/Akt and the mammalian target of rapamycin (PI3K/AKT/mTOR) pathway, receptor tyrosine-kinases (RTKs), DNA damage repair (DDR) and cell cycle progression, DNA-histone modifiers, and immune checkpoints. 

## 2. Small-Molecule Inhibitors for Endometrial Cancer Therapy

### 2.1. Targeting PI3K/AKT/mTOR Pathway

Increased PI3K/AKT/mTOR molecular pathway activity is seen in many different human cancers, probably because this pathway promotes cellular growth, metabolism, proliferation, survival, migration, apoptosis, and angiogenesis [7]. Among solid tumours, EC presents the highest alteration rate of the PI3K/AKT/mTOR pathway, being altered specifically in 92% of type I and 60% of type II ECs [3]. Usually, PI3K/AKT/mTOR pathway overactivation occurs as a result of overexpression of upstream tyrosine kinase growth factor receptors, phosphatase and tensin homolog (PTEN)loss-of-function (mutated in 67–84% of EC type I and 3–22% of EC type II), amplification or mutation in PI3KCA (38–55% of EC type I and 10–47% of EC type II), PIK3R1 (21–43% of EC type I and 12–17 of % of EC type II), and AKT genes (2% of EC type I) and elevated expression of mTOR (7% of EC type I) [8,9]. Overall, these data underline that the PI3K/AKT/mTOR pathway has a crucial role in EC pathogenesis, despite the slight differences in the frequency and types of alterations found in the two main histological tumour types. PTEN mutations seem to have a critical role in type I EC pathogenesis, while those found in mTOR may be primarily involved in the onset of type II EC.

**Figure 1:**
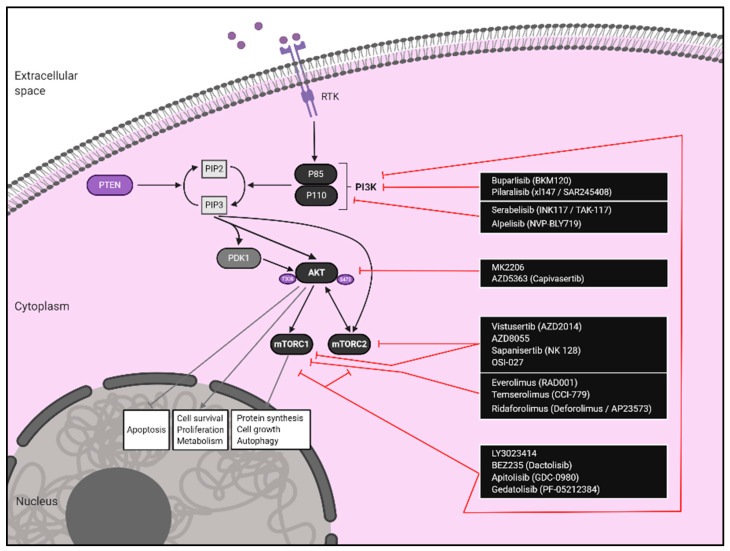
PI3K/AKT/mTOR signalling pathway SMIs for the treatment of endometrial cancer. Current SMIs under clinical investigation and their target molecules are displayed. Abbreviations: RTK, Receptor Tyrosine Kinase.

The high percentage of alterations observed in the PI3K/AKT/mTOR pathway in EC encourages the investigation towards the development of specific targeted small inhibitors acting on the three main molecular steps of this pathway (PI3K/AKT/mTOR). In particular, the inhibitors of the PI3K/AKT/mTOR pathway that will be deeply reviewed in this section fall into four main categories: mTOR inhibitors, PI3K inhibitors, AKT inhibitors, and dual mTOR/PI3K inhibitors (Figure 1). 

#### 2.1.1. mTOR Inhibitors

The first studies in EC using SMI were focused on mTOR and, in particular, on Rapamycin analogues. Rapamycin and other rapalogs are classified as first-generation mTOR inhibitors, which suppress the mTORC1 but not the mTORC2 [10]. Three of these compounds—Everolimus, Temsirolimus, and Ridaforulimus—have completed phase II clinical trials tested as monotherapy in EC and are currently being evaluated in combinational trials.

Everolimus (RAD001), a specific inhibitor of mTORC1, was the first oral mTOR inhibitor studied in EC. A phase II study assessed 28 patients with persistent or recurrent type I endometrial disease. Response was determined by tumour assessments from radiological tests or physical examination using Response Evaluation Criteria in Solid Tumours (RECIST). Complete response (CR) was considered as the disappearance of all target and non-target lesions; partial response (PR) was indicated when target measurable lesions were reduced by at least 30%; progressive disease (PD) was reported when the target lesions increased 20% in size and/or new lesions appeared in the first weeks of treatment; and stable disease (SD) included any condition not meeting the above criteria. In this phase II study, no PR or CR were observed, but 43% showed SD at the first evaluation time (8 weeks) (NCT00087685) [11]. 

In another phase II trial testing the efficacy of Everolimus as a single agent on advanced or metastatic EC refractory to one or two previous chemotherapy regimens, the results showed that 36% of patients had a non-progressive disease in the first 3 months after treatment, 9% of patients presented PR, and 27% showed SD at 6 months. Still, no CR was observed (NCT00870337) [12]. Better results were obtained by the combination of Everolimus and the aromatase inhibitor Letrozole, which in an open phase II clinical trial obtained 9 CR and 2 PR out of 38 patients enrolled in the study (NCT01068249) [13]. A similar clinical trial, studying the combination of Everolimus, Letrozole, and Metformin, enrolling 62 patients also obtained comparable results. Data showed that patients with advanced EC present 28% PR and 22% SD. Interestingly, women presenting endometrioid histology and catenin (cadherin-associated protein), beta 1 (CTNNB1) mutations had a better response to this combinational therapy (NCT01797523) [13,14]. This could be explained by the fact that the stabilization of β-catenin, derived from mutations in CTNNB1, could result in the activation of mTORC1. Thus, Everolimus treatment would be beneficial for those patients [13].

Everolimus was evaluated with another aromatase inhibitor, Anastrozole, in a phase II trial. The combination showed promising results with a CR in 17% of EC patients. However, extensive research is imperative due to the small number of EC patients (*n* = 6) enrolled in this trial (NCT01197170) [15].

A phase II trial of intravenous Temsirolimus (CCI-779) in patients with recurrent and/or metastatic EC evaluated both patients who were treated with chemotherapy and patients who were not. Temsirolimus showed 14% of PR and 69% of SD among the chemotherapy-naive group. On the other hand, there were 4% of PR and 48% of SD among patients in the chemotherapy-treated group. PTEN expression, defined by immunochemistry, failed to predict response to this inhibitor (NCT00072176) [16,17]. This modest response encouraged investigators to study Temsirolimus in combination with conventional chemotherapy. A phase II study investigating this showed that Temsirolimus with Paclitaxel and Carboplatin demonstrated good tolerability, but there were no differences in terms of response or SD compared to the historical data of Paclitaxel and Carboplatin treatment (NCT00977574) [18]. In the randomized phase II GOG248 trial, the authors compared intravenous Temsirolimus regimen versus the combination of Temsirolimus plus a hormonal therapy alternating Megestrol Acetate and Tamoxifen. The combination arm was closed early because of an excess of venous thrombosis. However, the accrual of patients to single-agent Temsirolimus therapy continued. The study concluded that patients enrolled in a single Temsirolimus arm presented 28% PR. In the second stage of the study, a total of 50 patients were treated on the same arm; 22% of PR was observed and 26% presented SD (NCT00729586) [19]. Another phase II trial testing the benefit of Temsirolimus in combination with other SMIs, such as Bevacizumab, has been conducted. However, the combinational regimen of Temsirolimus plus Bevacizumab was associated with significant toxicity (NCT00723255) [20].

Results extrapolated from clinical trials on Ridaforolimus (deforolimus, AP23573) in patients with recurrent or persistent EC and disease progression after one or two lines of chemotherapy showed an 11% of PR and 18% of SD (NCT00122343) [21]. By contrast, in another phase II trial, Ridaforolimus was evaluated in 34 patients with recurrent or metastatic EC who did not receive previous systemic chemotherapy as a single agent. The results showed a modest activity of Ridaforolimus, with an observed PR of 9% and 18% SD. As also seen in clinical trials with Temsirolimus, PTEN mutational status did not correlate with PR or SD (NCT00770185) [22]. In the same line, Oza et al. reported on a randomized phase II trial of Ridaforolimus in women with advanced EC who had progressive disease following one or two lines of chemotherapy and no hormonal therapy. The trial compared Ridaforolimus treatment versus progestin or systemic chemotherapy. Ridaforolimus treatment was discontinued as a result of adverse events in 33% of patients compared to 6% in the progestin or chemotherapy group. The authors concluded that although Ridaforolimus showed encouraging activity in advanced EC, its activity was associated with significant toxicity (NCT00739830) [23].

In the last years, dual mTOR inhibitors (known also as second-generation inhibitors) have been developed. These small inhibitors can suppress both the mTORC1 and mTORC2 and their main advantage is the considerable decrease of AKT phosphorylation and better inhibition of mTORC1. Those inhibitors are developed to have an increased therapeutic effect and less resistance [9]. Preclinical studies analysing the effect of second-generation mTOR inhibitors, such as Vistusertib (AZD2014) and AZD8055, have resulted in dose-dependent growth inhibition and apoptosis in a variety of EC cell lines [24,25]. Currently, various second-generation mTOR inhibitors, such as Vistusertib (AZD2014), Sapanisertib (INK 128), and OSI-027, are in early stage clinical trials in EC (NCT02730923, NCT02725268, and NCT02208375). 

#### 2.1.2. PI3K Inhibitors

PI3K inhibitors are either pan-PI3K inhibitors (which inhibit all four classes of PI3Ks) or isoform-selective PI3K inhibitors. Buparlisib (BKM120) is an oral pure PI3K inhibitor with broad antitumor activity in preclinical studies and in a phase I clinical trial [26]. Buparlisib was tested in a phase II study including 40 patients with advanced or recurrent EC. Unfortunately, none of them presented an objective response (OR), which is a measure that compiles any response given by the patient, either complete or partial. Moreover, a huge percentage of patients (87%) experienced adverse events. Thus, the clinical trial was stopped before the end of recruitment due to toxicity (NCT01397877) [27]. Pilaralisib (XL147 or SAR245408) is an oral pan-class I PI3K inhibitor. This drug was evaluated as monotherapy in a phase II open-label study that recruited 67 patients with advanced or recurrent EC who had received one or two prior chemotherapy regimens. The results showed that Pilaralisib presents a favourable safety profile, although it has minimal antitumor activity (3% of PR and 37.3% of SD) (NCT01013324) [28]. Pilaralisib was studied in combination with a Paclitaxel and Carboplatin regimen in a phase I clinical trial. The results obtained did not appear to enhance the antitumoral efficacy of Pilaralisib in monotherapy (NCT00756847) [29].

An alternative strategy to pan-PI3K small inhibitors is to specifically target the PI3K p110 catalytic isoform, which, because of its importance and differentiated role, presents the theoretical advantage of a better safety profile. P110α selective inhibitors, such as Serabelisib (INK117 or TAK-117) and Alpelisib (NVP-BLY719), have shown preclinical antitumoral activity in EC cell lines harbouring PTEN and PI3KCA mutations [30]. The first in-human phase Ia study using Apelisib has shown a tolerable safety profile and encouraging preliminary activity in patients since among the three EC patients included in the study, one had CR and another one PR (NCT01219699) [31]. At present, a phase Ib/II study of Serabelisib in combination with Canagliflozin in patients with advanced solid tumours, including EC, is ongoing (NCT04073680).

#### 2.1.3. Dual mTOR/PI3K Inhibitors

An important limitation to the mTOR- and PI3K-independent small inhibitors is that they are likely to present numerous signalling feedback loops. Thus, dual mTOR/PI3K inhibitors are designed to bind the ATP-binding site of both class I PI3Ks and mTORC1/2 and should lead to a more complete suppression of the PI3K/AKT/mTOR pathway. Among the most advanced dual inhibitors, LY3023414 has demonstrated a tolerable safety profile as a single agent in patients with advanced cancers including EC in a phase I clinical trial (NCT01655225) [32]. Additionally, this compound was tested in an open-label phase II study that included 28 patients with advanced EC harbouring activating mutations in the PI3K/AKT/mTOR pathway. The data obtained showed a modest activity of 14.3% of PR and 35.7% of SD (NCT02549989) [33]. 

BEZ235, also known as Dactolisib, is a potent, highly specific, and selective inhibitor of class I PI3K that has proven great sensitivity in preclinical studies using EC cell lines [34] and reduced tumour growth in EC xenograft models [35]. However, phase I trials administering BEZ235 as monotherapy in patients with advanced solid tumours (including EC) did not observe objective responses (NCT01343498) [36]. Finally, a clinical trial examining BEZ235 in combination with Everolimus showed limited clinical efficacy and tolerance (NCT01508104) [37]. 

A phase I clinical trial of Apitolisib (GDC-0980) demonstrated favourable pharmacokinetics and evidence in patients with advanced solid tumours (NCT00854152) [38]. Nonetheless, a multicentre, single-arm, and open-label phase II study of Apitolisib in patients with recurrent or persistent EC showed a relative narrow therapeutic index (5% PR) and poor tolerability, especially in diabetic patients (NCT01455493) [39]. 

Gedatolisib (PF-05212384) is another potent dual PI3K/mTOR inhibitor that has proved effective in two clinical trials in patients with recurrent EC following platinum-containing chemotherapy. Gedatolisib has shown manageable toxicity and was active as a single agent since out of the 38 advanced EC patients enrolled in the phase II study, 16% presented PR and 37% SD (phase I: NCT01347866 phase II: NCT01420081) [39,40]. At the moment, NCT03065062 and NCT02069158 trials have been organized for testing Gedatolisib combined with Palbociclib or systemic chemotherapy in different solid tumours, including EC, although definitive results of these studies are not yet available.

#### 2.1.4. AKT Inhibitors

Few preliminary data are available on the clinical use of SMI targeting AKT in EC. A two-arm PI3KCA mutation stratified phase II trial on MK2206 (allosteric inhibitor of AKT) revealed a high number of toxicities and limited efficacy in recurrent EC patients, regardless of PI3KCA mutation status. Interestingly, modest clinical activity was found in serous histological patients, warranting further studies prompting a histologic-specific expansion (NCT01307631) [41].

AZD5363 (Capivasertib) is a selective inhibitor of AKT that has exhibited good results and those were strongly correlated with the presence of PIK3CA mutations in preclinical EC models [42]. A phase I trial showed that AZD5363 was well-tolerated and achieved robust AKT modulation in EC tumours (NCT01226316) [43]. Moreover, preliminary results from a phase I in recurrent EC patients that evaluated the combination of AZD5363 and Olaparib have shown 50% of PR (NCT02208375) [44]. These results encourage the undertaking of a subsequent phase II trial to evaluate the combination of Olaparib and AZD5363 in recurrent EC. 

In addition, other inhibitors that indirectly modulate the PI3K/AKT/mTOR pathway, such as ABTL0812, are under investigation. In particular, ABTL0812 is being studied in a phase II clinical study on recurrent and metastatic EC patients (NCT03366480) after completing a promising phase I study (NCT02201823) that showed a highly safety profile and the second longest SD in an EC patient included in the study [45]. 

### 2.2. Targeting Receptor Tyrosine Kinases

RTKs are key actors in the normal physiology of cells, as they play critical roles by regulating a wide spectrum of processes, such as growth, motility, differentiation, and metabolism [46]. All RTKs share a similar protein structure containing an extracellular ligand-binding domain, a single transmembrane helix, and an intracellular domain with a tyrosine kinase domain (TKD) and a carboxyl-terminal tail [47]. Abnormal RTK activation in human cancers is mediated by four principal mechanisms: gain-of-function mutations, genomic amplification, chromosomal rearrangements, and/or autocrine activation [46]. Due to the RTK dysregulation being closely associated with cancer development and progression, a great effort is being made to develop novel molecules able to target RTKs. Many of these RTK inhibitors target the ATP-binding site of the intracellular TKD, impairing downstream signal transduction. In EC, RTKs are frequently mutated (Table 1) [3].

Here, we will analyse novel RTK inhibitors involved in EC treatment, by grouping them in different categories according to the RTKs that they target: SMIs of the epidermal growth factor receptor (EGFR), SMIs of the mitogen-activated protein kinase (MAPK) pathway, pan-TK inhibitors targeting the fibroblast growth factor receptor (FGFR), pan-TK inhibitors targeting angiogenesis (vascular endothelial growth factor receptor (VEGFR)), and pan-TK inhibitors targeting other RTKs.

#### 2.2.1. EGFR Inhibitors

Afatinib is a potent, selective, and irreversible ErbB family blocker that covalently binds to the TKD of the receptor and blocks signalling from all ErbB family members, causing tumour growth inhibition and regression [50]. Many preclinical studies showed the efficacy of Afatinib treatment towards epidermal growth factor receptor 2 (HER2)-amplified uterine serous carcinoma (USC) models, resulting in the abrogation of cell survival, inhibition of HER2/neu autophosphorylation, and pathway blockage [48,49]. However, there are few reports showing Afatinib efficacy within EC patients. A case report showed the clinical benefit of Afatinib treatment in an endometrioid EC stage IIIC patient refractory to multiple lines of chemotherapy [51]; similarly, an ongoing phase II clinical trial (NCT02491099), which is estimated to be concluded by 2023, is also evaluating PFS on HER2-positive USC patients.

Erlotinib reversibly binds to EGFR TKD, specifically at the adenosine triphosphate (ATP)-binding site [52]. Oza et al. designed a multinomial two-stage phase II study (NCT00030485) to evaluate the activity of Erlotinib in recurrent or metastatic advanced EC women, who were chemotherapy naive and had received up to first-line hormonal therapy. Even though treatment was well tolerated, they observed a modest response rate, 12.5%, with 46.9% of the patients showing SD. Of note, from the 32 patients enrolled, only 19 were EGFR positive; thus, it was not possible to associate Erlotinib’s response to EGFR mutation or amplification [53]. 

Gefitinib acts similarly to Erlotinib by selectively blocking the ATP-binding site of EGFR TKD. Results from a phase II clinical trial (NCT00027690) evaluating the efficacy and safety of Gefitinib in patients with persistent/recurrent EC showed that treatment was well tolerated but lacked sufficient efficacy. From the 26 recruited patients, only 4 (15%) experienced PFS ≥ 6 months, and 1 (3.8%) had a CR [54]. 

Lapatinib is an HER2 and EGFR TK inhibitor, which binds to TKD, preventing receptor autophosphorylation upon ligand binding [55]. Chu et al. performed a phase I open-label study to determine the safety, optimally tolerated regimen (OTR), pharmacokinetics, and clinical activity of Lapatinib in combination with Letrozole in patients with advanced breast cancer and other solid malignancies, including two patients with EC. One of the EC patients experienced a PR after dual treatment that lasted 161 days [56]. Finally, the Gynaecologic Oncology Group performed a phase II trial (NCT00096447) to evaluate the efficacy and safety of the Lapatinib in persistent/recurrent EC and associated it to EGFR mutations. They found an insufficient overall clinical response with only 3 out of 31 patients having a PFS ≥ 6 months, 3.2% PR, 22.5% SD, and 67.7% PD. Interestingly, they were able to identify EGFR mutations, L688F, K754E, and E690K, with only E690K being associated with a Lapatinib-responder patient [57].

#### 2.2.2. MAPK Inhibitors

Selumetinib is a non-ATP-competitive MAPK kinase 1 and 2 (MEK-1 and MEK-2) inhibitor [55,56]. A phase II single-arm open-label study (NCT01011933) demonstrated that Selumetinib was well tolerated for women with recurrent EC but lacked efficacy [58].

#### 2.2.3. Pan-RTK Inhibitors Targeting FGFR

Dovitinib is a potent multiple-RTK inhibitor that targets the FGF, VEGF, and PDGF pathways. A multicentre, open-label, non-randomized phase II trial (NCT01379534) focused on evaluating the safety and efficacy of Dovitinib as a second-line treatment in patients with advanced and/or metastatic EC, and revealed that 32% of the FGFR2-mutated patients presented a PFS of 18 weeks and median overall survival (OS) of 20 months compared to 29% PFS and 9 months OS observed in FGFR2 non-mutated patients [59]. Another multi-targeted TK inhibitor is Brivanib, which targets VEGFR and FGFR. Brivanib was evaluated in recurrent or persistent EC and was well tolerated. However, only 18.6% of recruited patients had PR or CR in the phase II trial (NCT00888173) [60]. In this study, the authors suggested that apparently endometrioid and mixed epithelial subtypes would benefit from Brivanib therapy compared to serous histology. Lastly, Ponatinib is a novel multi-target TK inhibitor, particularly effective against T315I mutation of ABL1, but not restricted since it also inhibits VEGFR, PDGFR, FGFR, EPH receptors, and SRC kinases members, among others. This drug is currently being tested in a clinical trial (NCT01888562) focused on recurrent or persistent EC carrying FGFR mutations. 

#### 2.2.4. Pan-RTK Inhibitors Targeting Angiogenesis (VEGFR)

Cabozantinib is a non-specific TK inhibitor, acting on VEGFR-1, -2, and -3; KIT; and other kinases [61]. Cabozantinib was evaluated in a single-arm phase II clinical trial (NCT01935934) to test its efficacy and toxicity as a second-line treatment in women with advanced EC. They observed activity of Cabozantinib in both serous and endometrioid EC patients, with 6-month PFS rates of 29% to 40%, a response rate of 12% to 14%, respectively, and acceptable toxicity (17%). Nonetheless, most patients discontinued therapy due to disease progression [62]. Currently, two ongoing additional clinical trials are testing Cabozantinib in EC. The first one is a randomized phase II study (NCT03367741) in which patients with advanced, recurrent, or metastatic EC undergo Cabozantinib treatment in combination with the immunotherapeutic Nivolumab. The second is a phase Ib dose-escalation study (NCT03170960) using single or combined treatment of Cabozantinib and the immunotherapeutic Atezolizumab in patients with locally advanced or metastatic solid tumours, including EC. At present, no results have been reported yet for either of the clinical trials.

Cediranib is an orally available, highly potent, and selective VEGF signalling inhibitor of all members of the receptor [63]. A phase II study (NCT01132820) in 48 recurrent/persistent EC demonstrated that Cediranib administered as a single agent had 12.5% PR, a median PFS of 3.65 months, and median OS of 12.5 months [64]. Unfortunately, almost 30% of the patients discontinued treatment due to toxicity. Another phase II trial (NCT03660826) studied the effect of the combination of Olaparib and Cediranib in patients with recurrent, refractory, or metastatic EC. Similarly, another three-arm randomized phase II clinical trial (NCT03570437) is being performed to understand if the same combination represents a better therapeutic strategy to Paclitaxel in advanced-EC patients. 

Nintedanib is another SMI, competitive, triple angiokinase inhibitor that targets multiple RTKs, including PDGFR-α and -β, FGFR1,2,3, VEGFR1,2,3, and FLT3, by binding to the ATP-binding pocket and inhibiting their activity [62,63]. A phase II clinical trial (NCT01225887) in 32 advanced, recurrent, or metastatic EC patients reported that none of them had a CR and only 3 (9.3%) patients showed PR [65]. The effectiveness of Nintedanib in combination with conventional chemotherapy is also being evaluated in a randomized phase II study (NCT02730416), which focuses on patients with primary advanced stage, or with the first relapse of EC. No data is available till now.

Sunitinib is a small-molecule multi-targeted RTK inhibitor with activity against PDGFR, VEGFR, and KIT, among others. In this regard, Castonguay et al. reported a modest activity of Sunitinib in women with recurrent EC that were recruited in a phase II study (NCT00478426) to assess the efficacy and tolerability of Sunitinib. In this study, 30% of patients showed a 6-month PFS and 21% a 12-month PFS [66]. The authors failed to identify clinico-pathological parameters linked to patients who would benefit from this therapy.

The RTK inhibitor Lenvatinib targets kinases implicated in pathogenic angiogenesis, tumour growth, and cancer progression, such as VEGFR1,2,3, PDGFR-α, FGFR1,2,3,4, KIT, and RET [67]. There are many ongoing clinical trials testing Lenvatinib in EC. Vergote et al. reported the results from a single-arm phase II study (NCT01111461) that aimed to evaluate the efficacy and safety of Lenvatinib in patients with recurrent or advanced EC after the failure of first-line platinum-based therapy. They observed that the treatment had a modest antitumor activity as second-line therapy in this cohort, with 21% of patients having an objective response, lasting 7.2–8 months, and a median PFS of 5.6 months and OS of 10.6 months [68]. On the other hand, Makker et al. designed a single-arm, phase Ib/II study (NCT02501096) to determine the maximum tolerated dose (MTD) for Lenvatinib in combination with Pembrolizumab and subsequently the safety and efficacy of the combination in solid tumours. They reported that the combinatory treatment showed antitumour activity in those patients with advanced recurrent EC, with an objective response in 35.5% (16/45) of patients with microsatellite-stable tumours [69]. As a result of these findings, they moved forward to a phase III clinical trial (NCT03517449) to evaluate Lenvatinib plus Pembrolizumab treatment in comparison to Doxorubicin or Paclitaxel in patients with advanced EC. This study is expected to conclude in 2022. In parallel, another two studies are ongoing: A randomized phase II trial (NCT03005015) that evaluates Lenvatinib compared to Doxorubicin, and NCT03884101, which compares the efficacy of Lenvatinib plus Pembrolizumab with respect to chemotherapy (Carboplatin-Paclitaxel), both in advanced and recurrent EC. 

#### 2.2.5. Pan-RTK Inhibitors of Other Targets

Dasatinib is an oral dual BCR/ABL and Src family TK inhibitor with activity against c-KIT, EPHA2, and PDGFRβ [70]. Few studies are reported on Dasatinib administration in EC. Duska et al. designed a phase 0 trial (NCT01482728) to study drug- and dose-specific changes in Src levels and activity, by analysing the tumour, normal adjacent tissue, and blood from newly diagnosed EC patients previous to surgery. They found that Dasatinib entered the solid tumour tissue and modulated Src activity. Similarly, they demonstrated that Dasatinib concentrations were higher in tumour tissue than in adjacent normal tissue, providing for the first time an insight into the biological effects and potential therapeutic efficacy of Dasatinib in EC [71]. Nowadays, there is an ongoing phase I trial (NCT01440998), which aims to evaluate Dasatinib, Paclitaxel, and Carboplatin treatment in patients with stage III-IV or recurrent EC; there is also a phase II clinical trial (NCT02059265) on patients with recurrent or persistent ovarian, fallopian tube, endometrial, and peritoneal cancers. 

### 2.3. Targeting DNA Damage Repair and Cell Cycle Progression

Compromised DDR pathways are found in EC, resulting in uncontrolled tumour cell proliferation and accumulation of genomic alterations [72]. Moreover, around 15% of serous-like EC display homologous recombinational repair-deficient (HRR-deficient) genomic signatures [73] and loss of function of mismatch repair pathways are present in 28.6% of low-grade and 54.3% of high-grade endometrioid EC [74], suggesting that these patient populations may be good candidates for DDR small inhibitor therapies.

#### 2.3.1. Poly ADP Ribose Polymerase (PARP) Inhibitors (PARPi)

As PARPi have emerged as a novel therapy for advanced cancers [75], several clinical trials are currently evaluating the use of Olaparib, Niraparib, or Rucaparib, the three principal PARPi, in EC patients. Among others, Olaparib was evaluated in combination with cediranib and durvalumab in a phase I study. Promising results were obtained with 44% of PR and 33% of SD (NCT02484404) [76]. Nowadays, there are several active phase I/II clinical trials evaluating the use of PARPi in combination with other therapies, such as PI3K, PD-1, and VEGF inhibitors (NCT02208375, NCT01237067, NCT03572478, and NCT03476798), and some phase II clinical trials are recruiting patients to evaluate the responses to PARPi as monotherapy or in combination with other inhibitors, such as PI3K, PD-L1, histone deacetylases (HDACs), or ATR inhibitors (NCT04171700, NCT03016338, NCT03586661, NCT03951415, NCT03924245, and NCT04065269).

#### 2.3.2. New DDR Inhibitors (DDRi)

Recently, new DDRi are being developed against protein kinases that respond to different types of DNA damage and regulate specific cell-cycle transitions (ATM, ATR, DN-PKcs, CHK1, CHK2, and WEE1) [77]. In EC, the DNA-PK inhibitor LY3023414 was evaluated in a phase I clinical trial and demonstrated to be safe in patients with advanced cancer. A durable PR lasting 18 months was observed in a woman with EC (NCT01655225) [32]. Currently, a phase II study is ongoing to determine the effectiveness of this compound in patients with recurrent or persistent EC (NCT02549989). Another phase II study is also ongoing to test the efficacy of the ATR inhibitor AZD6738 and Olaparib in patients with EC (NCT04065269).

#### 2.3.3. Cyclin-Dependent Kinase Inhibitors (CDKi)

Cell-cycle regulators play important roles in EC proliferation and many of these are frequently mutated, such as p53, or have altered expression patterns, such as the loss of p16 expression or cyclins A, D, and E overexpression [74]. Thus, SMIs targeting cyclin-dependent kinase (CDK) activity offer an opportunity for therapeutic intervention in these EC subgroups, since CDKi recapitulate the effects of checkpoint activation [78]. Ongoing studies are evaluating the activity of a third-generation CDKi in EC. The majority of these are active phase I or II clinical trials, which study the combination of endocrine therapies with one of the three main CDKi, Palbociclib, Abemaciclib, or Ribociclib, which inhibit CDK4/6 (NCT02657928, NCT02730429, NCT04188548, NCT03643510, NCT03675893, NCT04049227, and NCT04393285). Moreover, there are other active clinical trials in phases I and II that assess the combination of these CDKi with PI3K inhibitors (NCT03065062 and NCT03008408).

### 2.4. Targeting DNA and Histone Modifiers

Epigenetic regulation by covalent post-translational histone or DNA modifications, such as acetylation and methylation, represent a reversible step that regulates many biological processes by gene expression regulation [79]. Several studies have identified DNA promoter hypermethylation in several tumour suppressor genes [77,78], altered histone methyltransferase activity [80], and high levels of HDACs [81] in advanced stages of EC. Thus, histone inhibitors and DNA modifiers might be promising therapeutic agents in EC.

Among different drugs, the HDAC inhibitor (HDACi) Entinostat (MS-275) completed a phase I clinical trial that evaluated the toxicity and efficacy in patients with EC, defining an optimal dose of a schedule of every 14 days for a future phase II study (NCT00020579) [82]. Currently, several active clinical trials are evaluating the efficacy of this drug in combination with other agents in EC patients (NCT03018249 and NCT03924245). An ongoing phase I/II clinical trial is evaluating the safety and efficacy of Tinostamustine (EDO-S101) HDACi in women with EC (NCT03345485). 

### 2.5. Targeting Immune Checkpoints

In order to escape from the immune system, EC cells can stimulate immune checkpoints by activating negative feedback mechanisms [83]. Currently, several active clinical trials are evaluating the efficacy of immune checkpoints inhibitors in EC, although none of them are completed yet. There are different active clinical trials with IDO1 inhibitors as a monotherapy (NCT03896113) or in combination with monoclonal antibody immunotherapies (NCT03277352, NCT02178722, and NCT04106414). Others phase I and II clinical trials are currently recruiting patients to evaluate the efficacy of PDL-1 inhibitors, arginase inhibitors, or agonists of adenosine receptors in combination with chemotherapy or immunotherapies (NCT04152018, NCT03314935, NCT03629756, NCT03454451, and NCT03671811); the results of these studies are eagerly awaited. 

## 3. Conclusions

Despite the advances in the early detection and surgical management of EC patients, a considerable number of metastatic and recurrent endometrial tumours are still diagnosed. Furthermore, women with advanced disease have few treatment options and poor prognosis. The combination of platinum salts (Cisplatin and Carboplatin) is the standard regimen for treating these patients. However, platinum salt regimens have not obtained satisfactory results in recurrent or persistent EC. Therefore, the search for alternative new drugs for patients with relapsed and metastatic EC is imperative. Over the past three decades, the success of SMIs over conventional chemotherapy has been clearly proven in different types of cancer. Small-molecule inhibitors overcome the limitations of traditional cancer therapies by targeting specific molecules dysregulated in cancer cells. SMIs successfully target membrane receptors and extracellular proteins and can also translocate through plasma membranes to impair intracellular proteins’ function turning treatment more specifically than conventional chemotherapy. Even though they have wide-spectrum activity, SMIs still present certain limitations regarding toxicity. Some of the most reported toxicities include hypoglycaemia, cardiac toxicity, immunosuppression, pneumonitis, cutaneous reactions, nausea, diarrhoea, colitis, and hepatotoxic effects. However, the benefits of SMI targeted therapy widely exceeds its drawbacks (completed clinical trials summarized in Table 2).

Cancer treatment with targeted therapies, such as SMIs, requires a detailed knowledge of tumour biology. Undoubtedly, in the last few years, our knowledge of EC biology has brought on the recognition of molecular pathways involved in endometrial tumorigenesis, leading to the identification of molecular targets for novel treatment strategies. In spite of it, as inferred from the studies presented and summarized above, there is no data showing irrefutable improvement of survival rates yet. The results could be explained by the fact that targeted therapies were generally administered as monotherapy, in over-treated patients, and there were few stratifications based on histology, and/or treatment-response biomarkers. 

Challenges for the future are diverse and complex, and concern primarily the identification of new pathways, the early identification of resistance, and the use of SMIs with old and new chemotherapeutic and targeted agents. Combinatory treatment emerged as a feasible alternative for EC patients. The advantages of using a combinatory treatment based on SMIs and conventional chemotherapy, radiation therapy or any other agents can be promising in the management of EC patients by directly hitting altered pathways of tumour cells, potentially reducing adverse side effects caused by high or long-lasting doses of chemotherapy/radiotherapy. Further studies must be performed to exploit this strategy. However, at present, we have excellent results with targeted immunotherapy, although still only in other tumour types; therefore, it would be desirable to test these new immuno-drugs in recurrent/persistent EC, in combination with SMIs. Currently, several studies are ongoing (summarized in Table 3) and results in the next years are highly anticipated. 

In conclusion, nowadays, there are few choices of useful treatment for persistent or recurrent EC patients; however, SMIs will be central in the development of new EC treatment strategies.

## Figures and Tables

**Table 1:** Mutation frequency of the most relevant RTKs in EC. Data obtained from cbioportal website (https://www.cbioportal.org) based on the following datasets: Endometrial Cancer (MSK) [48], Uterine Corpus Endometrial Carcinoma (TCGA, Firehose Legacy), Uterine Corpus Endometrial Carcinoma ([3], Uterine Corpus Endometrial Carcinoma [49].

RTK	Mutation Frequency
FGFR2	12.80%
KIT	7.20%
MET	6.80%
KDR	6.30%
PDGFRα	6.00%
ERBB2	5.10%
EGFR	4.90%
PDGFRβ	4.70%
ABL1	3.90%
BRAF	3.80%
BCR	3.60%
FLT1	6.90%
FLT4	4.90%
SRC	1.40%

**Table 2:** Completed clinical trials of SMIs in EC.

Drug	Target	Phase	ClinicalTrials.gov Identifier	*n*	Patient Inclusion	Monotherapy or Combination Therapy	Previous Chemotherapy	CR (%)	PR (%)	SD (%)	Toxicity (as % of Patients Affected by Serious Adverse Events)	Reference
***PI3K/AKT/mTOR SMI***												
Everolimus	mTORC1	II	NCT00087685	28	Recurrent EEC	Monotherapy	Yes	0	0	43	71.4	[11]
		II	NCT00870337	44	Metastatic EC	Monotherapy	Yes	0	9	27	45	[12]]
		II	NCT01068249	35	Recurrent EC	Combination (Letrozole)	Yes	26	6	9	n/a	[13]
		II	NCT01797523	54	EEC	Combination (Letrozole and Metformin)	Yes	0	28	22	n/a	[14]
		II	NCT01197170	50	Advanced or metastatic EC	Combination (Anastrozole)	Yes	4	6	24	n/a	[15]
Temsirolimus	mTORC1	II	NCT00072176	60 (33 A; 27 B)	Advanced or metastatic EC	Monotherapy	No (A)/ Yes (B)	0	14 (A); 4 (B)	69 (A); 48 (B)	33	[16]
		II	NCT00977574	115	Advanced or recurrent EC	Combination (Paclitaxel and Carboplatin)	No	n/a	n/a	n/a	50.4	[18]
		II	NCT00729586	50	Advanced EC	Monotherapy (A)/Combination (Megestrol Acetate and Tamoxifen) (B)	Yes (58%)/ No (42%)	4.8 (A); 0 (B)	14.3 (A); 20 (B)	61.9 (A); 70 (B)	36 (A); 61.9 (B)	[19]
		II	NCT00723255	49	Recurrent EC	Combination (Bevacizumab)	Yes	2	22.4	46.9	63.3	[20]
Ridaforolimus	mTORC1	II	NCT00122343	45	Recurrent EC	Monotherapy	Yes	0	11	18	51	[21]
		II	NCT00770185	31	Recurrent or metastatic EC	Monotherapy	No	0	8.8	52.9	n/a	[22]
		II	NCT00739830	130	Recurrent or metastatic EC	Monotherapy	Yes	0	0	35	n/a	[23]
Buparlisib	PI3K	II	NCT01397877	16	Advanced or metastatic EC	Monotherapy	Yes	0	0	25	75	[27]
Pilaralisib	PI3K	II	NCT01013324	67	EC	Monotherapy	Yes	3	3	37.3	n/a	[28]
		I	NCT00756847	52	Advanced EC	Combination (Paclitaxel and Carboplatin)	Yes	0	13.5	42.3	48.3	[29]
Alpelisib	PI3K	IA	NCT01219699	134	Advanced EC	Monotherapy	Yes	0.9	6	52	n/a	[31]
LY3023414	mTOR/PI3K	I	NCT01655225	38	Advanced or metastatic EC	Monotherapy	Yes	0	2,6	31.9	n/a	[32]
		II	NCT02549989	28	Advanced EC	Monotherapy	Yes	0	14.3	35.7	n/a	[33]
BEZ235	mTOR/PI3K	I	NCT01343498	33	Advanced EC	Monotherapy	Yes	0	0	45	39	[36]
		IB	NCT01508104	19	Advanced or metastatic EC	Combination (Everolimus)	Yes	0	0	9	n/a	[37]
Apitolisib	mTOR/PI3K	I	NCT00854152	56	Advanced EC	Monotherapy	Yes	0	7	77	n/a	[38]
		II	NCT01455493	56	Recurrent EC	Monotherapy	Yes	3.6	1.8	49.1	n/a	[39]
Gedatolisib	mTOR/PI3K	I	NCT01347866	81	Advanced or metastatic EC	Combination (Irinotecan (A)/PD-0325901 (B))	Yes	0	4.5 (A); 11(B)	n/a	29	[40]
		II	NCT01420081	38	Recurrent EC	Monotherapy	Yes	0	16	37	40	[84]
MK2206	AKT	II	NCT01307631	5	High-grade EC	Monotherapy	Yes	0	0	80	37.8	[41]
Capivasertib	AKT	I	NCT01226316	90	Advanced EC	Monotherapy	Yes	0	0	7	62	[43]
***Receptor Tyrosine Kinase SMI***												
Selumetinib	MEK1,2	II	NCT01011933	54	EC	Monotherapy	Yes	2	4	26	64	[58]
Erdafitinib	FGFR1,2,3,4, RET, CSF1, PDGFRα,β, KIT, VEGFR2	I	NCT01703481	188	EC	Monotherapy	Yes	---	11	16	64	[85]
Dabrafenib	BRAF, RAF1, SIK1, NEK11, LIMK1	I	NCT01954043	23	EC	Combination (Rabeprazole and Rifampin)	Yes	n/a	n/a	n/a	n/a	*
Anlotinib	VEGFR2,3	I/II	NCT02558348	12	EC	Monotherapy	Yes	n/a	n/a	n/a	n/a	*
Brivanib Alaninate	VEGFR2, FGFR2	II	NCT00888173	43	EC	Monotherapy	Yes	2.3	16.3	n/a	41.9	[60]
Cediranib Maleate	VEGFR2	II	NCT01132820	48	EC	Monotherapy	Yes	n/a	12.5	n/a	41.7	[64]
Dasatinib	PDGFR, SRC, EPH, BCR, ABL	I	NCT01440998	18	EC	Combination (Carboplatin and Paclitaxel)	No	n/a	n/a	n/a	n/a	*
		I	NCT01482728	12	EC	Monotherapy	No	n/a	n/a	n/a	n/a	[71]
Erlotinib hydrochloride	EGFR	II	NCT00030485	32	EC	Monotherapy	Yes	0	12.5	46.8	87.9	[53]
Gefitinib	EGFR	II	NCT00027690	56	Recurrent EC	Monotherapy	Yes	3.8	n/a	26.9	73	[54]
Lapatinib ditosylate	EGFR, ERBB2	II	NCT00096447	30	EC	Monotherapy	Yes	0	3.3	23.3	33.3	[57]
Lenvatinib	VEGFR1,2,3, PDGFRα, FGFR1,2,3,4, KIT, RET	II	NCT01111461	133	EC	Monotherapy	Yes	1.5	19.5	23.3	49	[68]
Nintedanib	VEGFR1,2,3, PDGFRα,β, FGFR1,2,3, SRC	II	NCT01225887	32	EC	Monotherapy	Yes	0	9.4	21.9	43.7	[65]
Perifosine	MAPK1, PRKCA	II	NCT00053794	17	EC	Monotherapy	Yes	n/a	n/a	n/a	n/a	[86]
Vatalanib	VEGFR1,2,3	I	NCT00268918	24	EC	Combination (Docetaxel)	Yes	n/a	n/a	n/a	n/a	*
Sunitinib Malate	VEGFR1,2,3, PDGFRα,β, KIT	II	NCT00478426	34	EC	Monotherapy	Yes	0	18.2	18.2	90.9	[66]
		II	NCT00474994	53	EC	Monotherapy	Yes	0	2.1	43.7	17	*
Dovitinib	FGF, VEGF, PDGF	II	NCT01379534	53	Advanced EC	Monotherapy	Yes	0	11.3	45.3	56.6	[59]
Trametinib	MEK1,2	I	NCT01138085	240	EC	Monotherapy	Yes	n/a	n/a	n/a	n/a	*
***DNA Damage Repair SMI***												
Olaparib	PARP	I	NCT02484404	9	EC	Monotherapy	Yes	n/a	44	33	n/a	[76]

Abbreviations: CR, complete response; PR, partial response; SD, stable disease; NM, not mentioned; n/a, not applicable; EC, endometrial cancer; EEC, endometrioid endometrial cancer; *n*, total amount of patients included to the clinical trial. *: Completed clinical trial with unpublished results. Data available at www.clinicaltrials.gov.

**Table 3:** Ongoing clinical trials of SMIs in EC.

Drug	Target	Phase	ClinicalTrials.gov Identifier	*n*	Patient Inclusion	Monotherapy or Combination Therapy	Expected Results
***PI3K/AKT/mTOR SMI***							
Vistusertib	mTOR	I/II	NCT02730923	72	Advanced EC	Combination (Anastrozole)	June 2020
		Ib	NCT02208375	159	Recurrent EC	Combination (Olaparib)	November 2021
Sapanisertib	mTOR	II	NCT02725268	241	Advanced or recurrent EC	Combination (Paclitaxel or MLN1117)	October 2020
Serabelisib	PI3K	Ib/II	NCT04073680	60	Advanced EC	Combination (Canagliflozin)	December 2021
Gedatolisib	mTOR/PI3K	I	NCT03065062	96	Recurrent or metastatic EC	Combination (Palbociclib)	January 2023
AZD5363	AKT	Ib	NCT02208375	159	Recurrent EC	Combination (Olaparib)	November 2021
***Receptor Tyrosine Kinase SMI***							
Afatinib, Adavosertib, Binimetinib, Capivasertib, Copanlisib, Copanlisib Hydrochloride, Crizotinib, Dabrafenib, Dasatinib, Defactinib, Erdafitinib, Ipatasertib, Larotrectinib, Nivolumab, Osimertinib, Palbociclib, Pertuzumab, GSK2636771, Sapanisertib, Sunitinib Malate, Taselisib, Trametinib, Trastuzumab, Ulixertinib and Vismodegib	EGFR	II	NCT02465060	6452	Advanced or recurrent EC	Monotherapy	June 2022
Anlotinib	VEGFR2-3	I/II	NCT02584478	48	Recurrent or metastatic EC	Combination (Carboplatin and Paclitaxel)	December 2020
		I/II	NCT04157491	23	Recurrent or metastatic EC	Combination (Anlotinib)	December 2022
BDTX-189	EGFR, ERBB2	I/II	NCT04209465	184	Advanced EC	Monotherapy	December 2023
Cabozantinib	MET, VEGFR2, RET	I/II	NCT03170960	1732	Advanced or metastatic EC	Combination (Atezolizumab)	December 2021
Cediranib	VEGFR2	II	NCT03570437	129	Advanced EC	Combination (Paclitaxcel and Olaparib)	September 2021
CPL304110	FGFR	I	NCT04149691	42	Advanced EC	Monotherapy	July 2020
Famitinib	EGFR, ERBB2, FLT1/3	II	NCT03827837	265	Advanced EC	Combination (Camrelizumab)	June 2021
Pemigatinib	FGFR1,2,3,4	I/II	NCT02393248	325	Advanced EC	Combination (Gemcitabine, Cisplatin, Pembrolizumab, Docetaxel, Trastuzumab and Retifanlimab)	December 2020
Rebastinib	SRC, LYN, FGR, HCK, KDR, FLT3, Tie-2, BCR, Abl1	I/II	NCT03601897	201	Advanced or metastatic EC	Combination (Paclitaxel)	November 2021
Afatinib	ERBB2	II	NCT02491099	50	Recurrent EC	Monotherapy	July 2028
Cabozantinib S-malate	MET, VEGFR2, RET,	II	NCT01935934	102	Recurrent or metastatic EC	Monotherapy	September 2020
Cediranib	VEGFR2	I	NCT01065662	50	Advanced EC	Combination (Temsirolimus)	April 2020
Dasatinib	PDGFR, SRC, EPH, BCR, ABL	II	NCT02059265	35	Recurrent EC	Monotherapy	November 2020
Lenvatinib Mesylate	VEGFR1,2,3, PDGFRα, FGFR1,2,3,4, KIT, RET	I	NCT02788708	26	Recurrent EC	Combination (Paclitaxel)	November 2020
Lenvatinib	VEGFR1,2,3, PDGFRa, FGFR1,2,3,4, KIT, RET	I	NCT03006887	6	EC	Combination (Pembrolizumab)	April 2020
		I/II	NCT02501096	357	EC	Combination (Pembrolizumab)	April 2020
		III	NCT03884101	720	Recurrent EC	Combination (Pembrolizumab, Paclitaxel and Carboplatin)	April 2023
		III	NCT03517449	780	Advanced EC	Combination (Pembrolizumab, Paclitaxel and Doxorubicin)	January 2023
Nintedanib	VEGFR1,2,3, PDGFRα,β, FGFR1,2,3, SRC	II	NCT02730416	148	Advanced EC	Combination (Carboplatin and Paclitaxel)	July 2022
Axitinib	VEGFR1,2,3	II	NCT04197219	26	Recurrent EC	Combination (Pembrolizumab)	December 2026
Crizotinib	MET, ALK	II	NCT04030429	40	Recurrent or metastatic EC	Monotherapy	December 2023
Lapatinib	EGFR, ERBB2, TUBB3	I	NCT01454479	24	Recurrent EC	Combination (Ixempra)	April 2021
***DNA Damage Repair and Cell Cycle Progression SMI***							
Olaparib	PARP	I/II	NCT02208375	159	Recurrent EC	Combination (Capivasertib and Vistusertib)	November 2021
		I	NCT01237067	77	Recurrent EC	Combination (Carboplatin)	December 2020
Rucaparib	PARP	I/II	NCT03572478	12	Recurrent or metastatic EC	Combination (Nivolumab)	December 2021
		II	NCT03476798	33	Recurrent EC	Combination (Bevacizumab)	February 2023
		II	NCT04171700	220	EC	Monotherapy	May 2022
Niraparib	PARP	II	NCT03016338	44	Recurrent EC	Monotherapy and combination (TSR-042)	December 2022
		I	NCT03586661	44	Recurrent EC	Combination (Copanlisib)	April 2022
Olaparib	PARP	II	NCT03951415	55	Advanced, recurrent or metastatic EC	Combination (Durvalumab)	July 2023
		I/II	NCT03924245	73	High-grade EC	Combination (Entinostat)	September 2025
		II	NCT04065269	40	Recurrent EC	Combination (AZD6738)	March 2023
LY3023414	DNA-PK	I	NCT01655225	156	Advanced or metastatic EC	Monotherapy and combination (Midazolam, Fulvestrant, Pemetrexed, Cisplatin, Abemaciclib and Letrozole)	December 2020
		II	NCT02549989	31	Recurrent EC	Monotherapy	September 2021
AZD6738	ATR	II	NCT04065269	40	Recurrent EC	Combination (AZD6738)	March 2023
Abemaciclib	CDK4/6	I	NCT04188548	186	Advanced or metastatic EC	Combination (LY3484356)	April 2023
Palbociclib	CDK4/6	II	NCT02730429	78	EEC	Combination (Letrozole)	December 2022
		I	NCT03065062	96	EC	Combination (Gedatolisib)	January 2023
Abemaciclib	CDK4/6	II	NCT03643510	25	Recurrent EC	Combination (Fulvestrant)	August 2021
		II	NCT03675893	40	Recurrent or metastatic EC	Combination (Letrozole)	May 2024
		I	NCT04049227	27	Recurrent EC	Combination (Letrozole)	July 2023
		II	NCT04393285	50	Advanced, recurrent or metastatic EC	Combination (Letrozole)	June 2023
Ribociclib	CDK4/6	II	NCT02657928	40	Recurrent EC	Combination (Letrozole)	July 2021
		I	NCT03008408	87	Recurrent or metastatic EC	Combination (Letrozole and Everolimus)	August 2022
***DNA and Histone Modifiers SMI***							
Entinostat	HDAC	I	NCT00020579	75	Metastatic EC	Monotherapy	March 2022
		II	NCT03018249	50	EEC	Combination (Medroxyprogesterone Acetate)	December 2020
		I/II	NCT03924245	73	High-grade EC	Combination (Olaparib)	September 2025
Tinostamustine	HDAC	I/II	NCT03345485	167	Advanced or metastatic EC	Monotherapy	July 2022
Celecoxib	IDO1	II	NCT03896113	48	EC	Monotherapy	June 2022
Epacadostat	IDO1	I/II	NCT03277352	10	Advanced or metastatic EC	Combination (INCAGN01876 and pembrolizumab)	July 2020
	IDO1	I/II	NCT02178722	444	EC	Combination (MK-3475)	August 2020
BMS- 986205	IDO1	II	NCT04106414	50	Recurrent EC	Combination (Nivolumab)	September 2021
***Immunocheckpoints SMI***							
PF-06801591	PD1	I	NCT04152018	104	Advanced or metastatic EC	Combination (PF-06940434)	March 2024
AB122	PD1	I	NCT03629756	44	Advanced EC	Combination (AB298)	September 2021
AB928	Adenosin receptor	I	NCT03629756	44	Advanced EC	Combination (AB298 and AB122)	September 2021
INCB001158	Arginase	I/II	NCT03314935	149	Advanced or metastatic EC	Combination (Oxaliplatin, Leucovorin, 5-Fluorouracil, Gemcitabine, Cisplatin and Paclitaxel)	October 2021
Ciforadenant	ADORA2A	I	NCT03454451	378	Advanced EC	Combination (Pembrolizumab and CPI-006)	December 2023
Pterostilbene	MUC1	II	NCT03671811	36	EC	Combination (Megestrol Acetate)	December 2020

Abbreviations: CR, complete response; PR, partial response; SD, stable disease; NM, not mentioned; EC, endometrial cancer; *n*, total amount of patients included to the clinical trial.
